# Dominance of the hypothalamus-pituitary-adrenal axis over the renin-angiotensin-aldosterone system is a risk factor for decreased insulin secretion

**DOI:** 10.1038/s41598-017-10815-y

**Published:** 2017-09-12

**Authors:** Makoto Daimon, Aya Kamba, Hiroshi Murakami, Satoru Mizushiri, Sho Osonoi, Kota Matsuki, Eri Sato, Jutaro Tanabe, Shinobu Takayasu, Yuki Matsuhashi, Miyuki Yanagimachi, Ken Terui, Kazunori Kageyama, Itoyo Tokuda, Shizuka Kurauchi, Shigeyuki Nakaji

**Affiliations:** 1 Department of Endocrinology and Metabolism, Hirosaki University Graduate School of Medicine, Hirosaki, Aomori, Japan; 2 Department of Social Medicine, Hirosaki University Graduate School of Medicine, Hirosaki, Aomori, Japan

## Abstract

How the association between the hypothalamus-pituitary-adrenal (HPA) axis and the renin-angiotensin-aldosterone system (RAAS) affects glucose metabolism were not well examined in a general population. Participants of the population-based 2015 Iwaki study were enrolled (n: 1,016; age: 54.4 ± 15.1 years). Principal component (PC) analysis identified two PCs: PC1 represented levels of the HPA axis (serum cortisol) and the RAAS (plasma aldosterone) as a whole, and PC2 represented the HPA axis relative to the RAAS (HPA axis dominance). We examined the association between these PCs and glucose metabolism using homeostasis model assessment indices of reduced insulin sensitivity (HOMA-R) and secretion (HOMA-β). Univariate linear regression analyses showed a correlation between PC2 and HOMA-β (β = −0.248, p < 0.0001), but not between PC1 and HOMA-β (β = −0.004, p = 0.9048). The correration between PC2 and HOMA-β persisted after adjustment for multiple factors (β = −0.101, p = 0.0003). No correlations were found between the PCs and HOMA-R. When subjects were tertiled based on PC2, the highest tertile was at greater risk of decreased insulin secretion (defined as the lower one third of HOMA-β (≤68.9)) than the lowest tertile after adjustment for multiple factors (odds ratio, 2.00; 95% confidence interval, 1.35–2.97). The HPA axis dominance is associated with decreased insulin secretion in a Japanese population.

## Introduction

Type 2 diabetes (hereafter diabetes) is a heterogeneous disorder of glucose metabolism characterized by both reduced insulin sensitivity and pancreatic β-cell dysfunction. A variety of factors are thus involved in the pathophysiology of diabetes. Glucocorticoids (GCs) appear to be one of such factors, since GCs have various effects on glucose metabolism including promotion of gluconeogenesis in liver, suppression of glucose uptake in skeletal muscle and adipocytes, promotion of lipolysis in adipocytes, and suppression of insulin secretion^1–7^. In clinical settings, an excess of GCs from GC administration or pathological conditions such as Cushing syndrome can lead to diabetes^1, 8, 9^. However, the effects of GCs at concentrations within the physiological range on glucose metabolism have not been well evaluated. Since serum cortisol concentrations are not generally increased in patients with obesity and diabetes^10, 11^, GCs within the physiological range do not seem to have a substantial impact on glucose metabolism. However, studies with inhibitors of 11β-hydroxysteroid dehydogenease-1 (HSD1), which converts inactive steroid cortisone into the active steroid cortisol in target tissues (liver and adipose), have shown some promising results in patients with diabeties^12–14^. Further, inhibition of GCs secretion was shown to have anorexigenic effects in rats^15^. These findings together indicate that higher GC concentrations are risk factors for diabetes.

Mineralocorticoids (MCs) are another type of hormone known to affect diabetes development, with an excess of MCs in pathological conditions such as primary aldosteronism (PA) resulting in decreased insulin sensitivity and secretion^12–19^. However, as for GCs, the effects of MCs within their physiological range on glucose metabolism have not been well evaluated.

In addition, to the associations reported between absolute concentrations of these hormones and diabetes, there is now a report of association between the relative concentrations of hormones and disease. An association between dominance of the hypothalamus-pituitary-adrenal (HPA) axis over the renin-angiotensin-aldosterone system (RAAS) (represented by the ratio of serum cortisol concentrations to plasma aldosterone concentrations (cortisol/aldosterone ratio)) and hypertension was recently reported^20^. Here, we investigate whether dominance of the HPA axis over the RAAS (i.e. cortisol/aldosterone ratio) rather than their absolute concentrations also affects diabetes.

Principal component (PC) analysis was used to discriminate between the absolute concentrations levels of serum cortisol and plasma aldosterone (PC1: levels) and dominance of the HPA axis over the RAAS (PC2: HPA axis dominance). We examined the association between these PCs and glucose metabolism using homeostatic model assessment (HOMA) indices, and found association between the HPA axis dominance and decreased insulin secretion in a general Japanese population. Our findings may help to identify patients at risk for future development of diabetes and those suitable for GC suppression therapies for metabolic disorders such as diabetes.

## Results

### Clinical characteristics of the study subjects

The clinical characteristics of subjects by gender are shown in Table 1. Mean ages were 52.1 ± 15.4 years for men and 55.8 ± 14.7 years for women. Most clinical characteristics were significantly different between men and women, including serum cortisol concentrations and plasma aldosterone concentrations, which were significantly higher in men than in women (serum cortisol concentrations: men10.4 ± 3.2 *vs*. women 8.5 ± 3.0 μg/dl; plasma aldosterone concentrations: men 130.8 ± 59.5 *vs*. women 122.8 ± 59.1 pg/ml).

**Table 1:** Clinical characteristics of the subject

Characteristics	Men	Women	p
Number	385	631	—
Age (yr)	52.1 ± 15.4	55.8 ± 14.7	0.0001**
Height (cm)	168.8 ± 6.7	155.0 ± 6.3	<0.0001**
Body weight (kg)	67.3 ± 10.7	53.6 ± 8.6	<0.0001**
Body mass index (kg/m^2^)	23.61 ± 3.25	22.30 ± 3.45	<0.0001**
Fat (%)	19.56 ± 5.83	29.13 ± 7.35	<0.0001**
Cortisol (μg/dl)	10.36 ± 3.15	8.45 ± 2.96	<0.0001**
Aldosterone (pg/ml)	130.8 ± 59.5	122.8 ± 59.1	0.0114*
Fasting plasma glucose (mg/dl)	81.8 ± 10.6	80.7 ± 10.4	0.1197
HbA1c (%)		5.66 ± 0.39	0.0096*
Fasting serum insulin:IRI (μ/ml)	4.39 ± 2.53	4.64 ± 2.44	0.1091
HOMA-R	0.90 ± 0.55	0.95 ± 0.60	0.0413*
HOMA-β	118.2 ± 117.6	137.0 ± 156.5	0.0005*
F-CPR (ng/ml)	1.08 ± 0.46	1.00 ± 0.39	0.0022*
CPI	1.33 ± 0.56	1.24 ± 0.42	0.0046*
IR(CPR)	0.269 ± 0.117	0.286 ± 0.111	0.0037**
Systolic blood pressure (mmHg)	125.5 ± 16.5	120.3 ± 17.4	<0.0001**
Diastolic blood pressure (mmHg)	78.1 ± 11.7	72.9 ± 11.2	<0.0001**
Total cholesterol (mg/dl)	203.9 ± 33.0	209.7 ± 34.6	<0.0001**
Triglyceride (mg/dl)	118.1 ± 80.8	81.6 ± 42.3	<0.0001**
HDL cholesterol (mg/dl)	61.3 ± 16.8	70.6 ± 16.6	<0.0001**
Serum uric Acid (mg/dl)	5.91 ± 1.18	4.33 ± 1.02	<0.0001**
Serum urea Nitrogen (mg/dl)	15.02 ± 3.77	14.42 ± 4.42	0.0275*
Serum creatinin (mg/dl)	0.81 ± 0.13	0.62 ± 0.12	<0.0001**
Hypertension: n (%)	154(40.0)	231(36.6)	0.2797
Hyperlipidemia: n (%)	169(43.9)	306(48.5)	0.1542
Diabetes: n (%)	14(3.6)	26(4.1)	0.7003
Obesity: n (%)	108(28.1)	118(18.7)	0.0005**
Drinking alcohol: n (%)	263(68.3)	178(28.2)	<0.0001**
Smoking (Never/ Past/ Current):n	147/126/112	503/70/58	<0.0001**

P < 0.05 and <0.01 are indicated by * and **, respectively. Data are mean ± SD or number of subjects (%).

The prevalence of hypertension (defined as blood pressure ≥140/90 mmHg or taking treatment for hypertension) were 40% for men and 36.6% for women, for hyperlipidemia (defined as total cholesterol ≥220 mg/dl, TG ≥150 mg/dl or taking treatment for hyperlipidemia) were 43.9% for men and 48.5 for women, and for diabetes (defined according to the 2010 Japan Diabetes Society criteria: i.e. fasting blood glucose (FBG) levels ≥126 mg/dl^21^ or in subjects whose FBG levels were not measured, glycated hemoglobin (HbA1c) levels ≥6.5%) were 3.6% for men and 4.1% for women. The prevalence of hypertension was similar to the 2010 national values for men and women aged 30–69 years reported by the Japanese government (50.8% and 33.7%, respectively)^22^. There are no reported national values for the prevalence of hyperlipidemia (using the same definition as this study), though our observed prevalence is similar to that reported in other areas of Japan^23–26^. We excluded 30 men and 18 women with diabetes (either on medication for diabetes and/or FBG level >140) from the present study, but with these individuals the prevalence of diabetes in men and women in our original sample (10.2% and 6.5%, respectively) was not substantially different from the Japanese national values (15.4% and 7.1%, respectively)^22^. The prevalence of obesity (defined as body mass index (BMI) ≥25) were 28.1% for men and 18.7% for women (those defined as BMI ≥30 were 4.4% and 2.5%, respectively), which were also not so different from the 2010 national values for men and women aged 30–69 years reported by the Japanese government (33.5% and 20.5%, respectively)^22^. More men drank alcohol and were current smokers than women (alcohol: men 68.3% *vs*. women 28.2%; smokers: men 29.1% *vs*. women 9.2%).

### Correlation between hormone concentrations (serum cortisol and plasma aldosterone) and HOMA indices (reduced insulin sensitivity and secretion)

Univariate correlations between clinical characteristics and HOMA indices (R and β) are shown in Table 2. Since many clinical characteristics such as gender, age, drinking alcohol, body fat percent, blood pressure, HbA1c, and serum lipid levels were found to be correlated with HOMA indices, these factors were used as covariates for adjustments in further analyses. Plasma aldosterone concentrations were correlated with both HOMA indices (R: β = 0.071, p = 0.0246; β: β = 0.168, p < 0.0001), however, these correlations became non-significant after adjustment for multiple other factors correlated with HOMA indices (Table 2). Conversely, although univariate regression analysis revealed no correlation between serum cortisol concentrations and HOMA-R (β = −0.022, p = 0.4763), it revealed a significant correlation between serum cortisol concentrations and HOMA-β (β = −0.173, p < 0.0001). This correlation remained significant after adjustment for multiple other factors correlated with HOMA-β including a C-peptide (CPR)-based index of reduced insulin sensitivity, IR(CPR), (β = −0.091, p = 0.0011).

**Table 2:** Factors correlated with HOMA indices

Characteristics	R		β	
	β	p	β	p
Sex(M/F)	0.064	0.0413*	0.010	0.0005**
Age (yr)	0.083	0.0083*	−0.416	<0.0001**
Height (cm)	−0.078	0.0131*	0.03	0.339
Body weight (kg)	0.35	<0.0001**	0.116	<0.0002**
Body mass index (kg/m^2^)	0.515	<0.0001**	0.122	<0.0001**
Fat (%)	0.471	<0.0001**	0.186	<0.0001**
Cortisol (μg/dl)	−0.022	0.4763	−0.173	<0.0001**
Aldosterone (pg/ml)	0.071	0.0246*	0.168	<0.0001**
Fasting blood glucose (mg/dl)	0.495	<0.0001**	−0.637	<0.0001**
HbA1c (%)	0.325	<0.0001**	−0.318	<0.0001**
Fasting serum insulin:IRI (μU/ml)	0.977	<0.0001**	0.439	<0.0001**
F-CPR (ng/ml)	0.807	<0.0001**	0.254	<0.0001**
CPI	0.807	<0.0001**	0.254	<0.0001**
IR(CPR)	−0.837	<0.0001**	−0.02	0.5142
Systolic blood pressure (mmHg)	0.17	<0.0001**	−0.166	<0.0001**
Diastolic blood pressure (mmHg)	0.202	<0.0001**	−0.065	0.0381*
Total cholesterol (mg/dl)	0.138	<0.0001**	−0.08	0.0112*
Triglyceride (mg/dl)	0.277	<0.0001**	0.063	0.0442*
HDL cholesterol (mg/dl)	−0.27	<0.0001**	−0.142	<0.0001**
Serum uric Acid (mg/dl)	0.163	<0.0001**	−0.011	0.7330
Serum urea Nitrogen (mg/dl)	0.024	0.4364	−0.245	<0.0001**
Serum creatinin (mg/dl)	0.015	0.6385	−0.031	0.3293
Hypertension: n (%)	0.175	<0.0001**	−0.172	<0.0001**
Hyperlipidemia: n (%)	0.204	<0.0001**	−0.033	0.2936
Diabetes: n (%)				
Obesity: n (%)	0.424	<0.0001**	0.092	0.0032**
Drinking alcohol: n (%)	−0.127	<0.0001**	−0.161	<0.0001**
Smoking (Never/ Past/ Current):n	−0.06	0.0558	0.081	0.0100

P < 0.05 and <0.01 are indicated by * and **, respectively. Data are mean ± SD or number of subjects (%).

### Correlation between HPA axis dominance and HOMA indices

**Figure 1:**
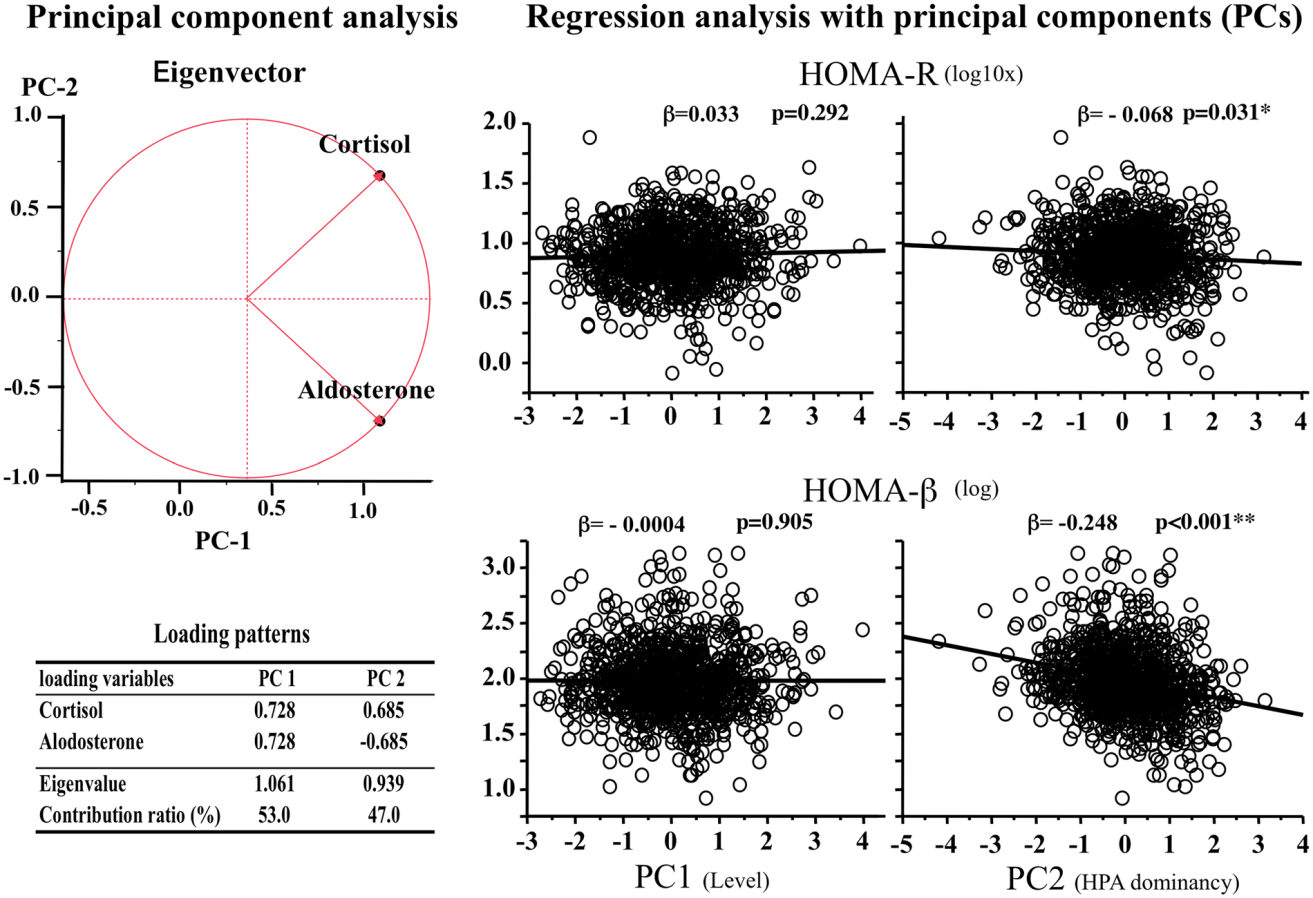
Correlation between principal components (PCs) generated with serum cortisol and plasma aldosterone concentrations and homeostasis model assessment (HOMA) indices. Eigenvectors and loading patterns of PCs are shown in the left panels. Correlations between PCs and HOMA indices are shown in the right panel with the correlation coefficient and the p value on each panel. Values of p < 0.05 are indicated by*.

We then discriminated between absolute hormone concentrations and their relative concentrations (dominance of the HPA axis over the RAAS) by PC analysis, which determined two PCs (PC1: levels; PC2: HPA axis dominance), and examined the correlations between these PCs and HOMA indices (Fig. 1). PC loadings for serum cortisol concentrations and plasma aldosterone concentrations were positive for PC1, while for PC2, PC loading was positive for serum cortisol concentrations, but negative for plasma aldosterone concentrations. These findings indicate that PC1 represents absolute hormone concentrations as a whole, while PC2 represents dominance of serum cortisol concentrations (the HPA axis) over plasma aldosterone concentrations (the RAAS). Univariate regression analysis revealed no correlations between PC1 and the HOMA indices, but revealed significant correlations between PC2 and both HOMA indices (R: β = 0.068, p = 0.0308; β: β = −0.248, p < 0.0001). After adjustment for multiple other factors correlated with the HOMA indices, the correlation between PC2 and HOMA-R became non-significant (β = 0.008, p = 0.6849), while the correlation between PC2 and HOMA-β remained significant (β = −0.101, p = 0.0003) (Table 3). Analyses using those with normal FBG levels (<100 mg/dl) showed similar results: the correlation between PC2 and HOMA-R was non-significant (β = 0.006, p = 0.7606), while the correlation between PC2 and HOMA-β was significant (β = −0.088, p = 0.0033). Further analyses stratified based on gender showed also similar results: the correlations between PC2 and HOMA-β were significant both in men (β = −0.101, p = 0.0206) and women (β = −0.095, p = 0.0090), while the correlations between PC2 and HOMA-R were non-significant both in men (β = 0.019, p = 0.5540) and women (β = 0.0004, p = 0.8663).

**Table 3:** Association of serum hormone concentrations with HOMA indices

	R				β			
	Univariate		Multiple factors adjusted^#1^		Univariate		Multiple factors adjusted^#2^	
	β	p	β	p	β	p	β	p
Cortisol	−0.022	0.4763	0.029	0.1348	−0.173	<0.0001**	−0.091	0.0011**
Aldosterone	0.071	0.0246*	0.009	0.6428	0.168	<0.0001**	0.047	0.0956
PC1 (levels)	0.033	0.2921	0.027	0.1554	−0.004	0.9048	−0.031	0.2697
PC2 (HPA dominance)	−0.068	0.0308*	0.008	0.6849	−0.248	<0.0001**	−0.101	0.0003**

Adjusted with #1age, gender, BMI, T-Cho, TG, HbA1c, dBP, Alcohol, SUN, CPI; #2 age, gender, %fat, T-Cho, HDL, HbA1c, sBP, Alcohol, SUN, IR(CPR). P <0.05 and <0.01 are indicated by * and **, respectively.

### Association of HPA axis dominance with decreased insulin secretion

**Figure 2:**
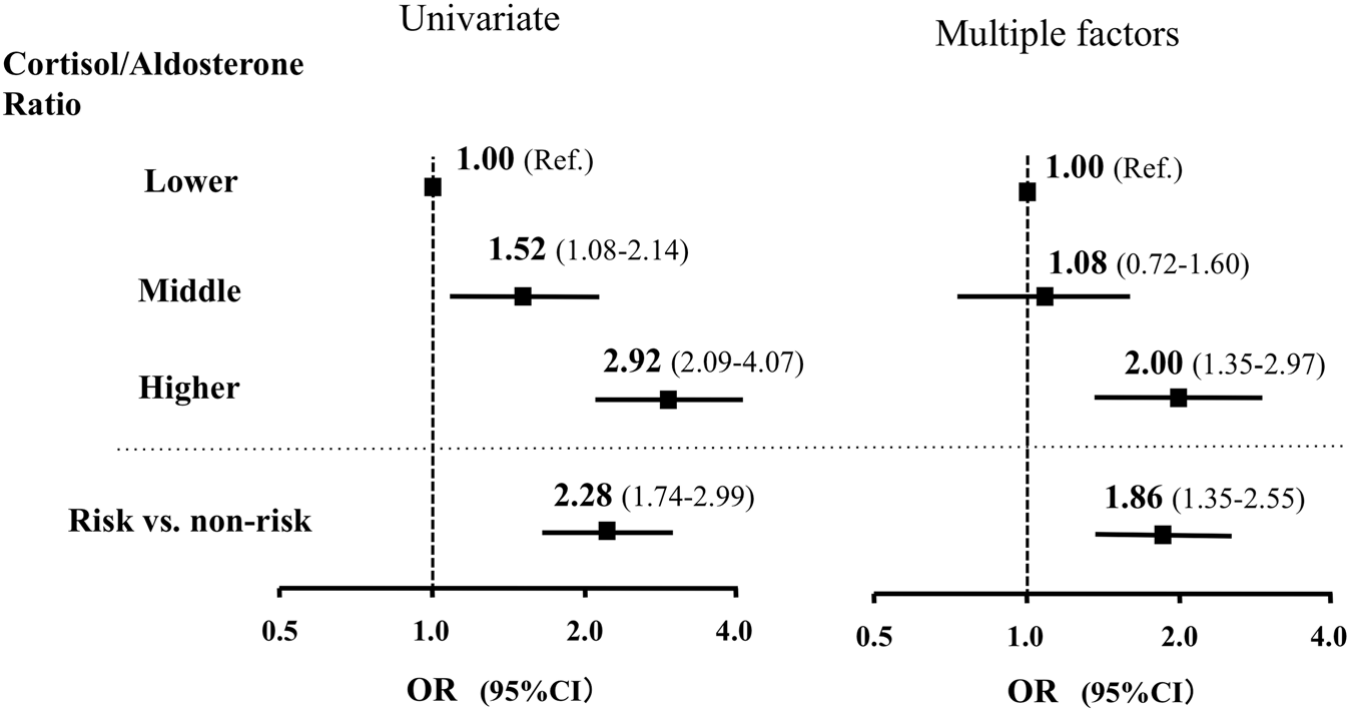
Risk for decreased insulin secretion. Values are odds ratios (OR)s with 95% confidence intervals (CI). Decreased insulin secretion was designated as the lower one third of homeostasis model assessmentβ (HOMA-β) values (≤68.9). Subjects were stratified into tertiles based on their ratio of serum cortisol (F, µg/dl) to plasma aldosterone (PAC, pg/ml) (higher > 0.095, middle 0.0610–0.095, lower ≤ 0.060). Subjects were also stratified into 2 groups (risk and non-risk) using the optimal cut-off value of the F/PAC ratio to predict decreased insulin secretion determined by receiver operating characteristic curve analysis (0.090 (µg/dl)/(pg/ml). Risks were adjusted for multiple factors including age,gender, %fat, T-Cho, HDL, HbA1c, sBP, Drinking alcohol, and SUN. Ref: reference.

Since the correlation between PC2 and HOMA-*β* observed was not strong, we further evaluated the correlation differently. Subjects were stratified into tertiles based on their PC2 scores i.e. the ratio of serum cortisol concentrations (μg/dl) to plasma aldosterone concentrations (pg/ml) (higher ≥0.095, middle 0.0610–0.095, lower ≤0.060). We then evaluated the risks of these tertiles for decreased insulin secretion, which we designated as the lower one third of HOMA-β (≤68.9) (Fig. 2). The higher cortisol/aldosterone ratio was a significant risk for decreased insulin secretion (odds ratio (OR): 2.92, 95% confidence interval (CI): 2.09–4.07). This risk remained significant after adjustment for multiple factors (age, gender, %fat, total cholesterol, HDL-C, HbA1c, systolic blood pressure, SUN, IR(CPR) and drinking alcohol) (OR: 2.00, 95% CI: 1.35–2.97). Furthermore, using the optimal cut-off value of the cortisol/aldosterone ratio to predict decreased insulin secretion determined by ROC curve analysis (0.090 (μg/dl)/(pg/ml) (area under the curve (AUC):0.632; sensitivity:0.509; specificity: 0.687)), those at risk had an OR of 1.86 (CI: 1.35–2.55) after adjustment for the factors listed above. Analyses using those with normal FBG levels and those stratified based on gender also showed similar results (Supplementary Tables 1 and 2).

Together, these results indicate that a higher cortisol/aldosterone ratio (i.e. HPA axis dominance), but not the hormone concentration per se, is a significant risk factor for decreased insulin secretion in this general Japanese population.

## Discussion

In this cross-sectional study of a general Japanese population, we used PC analysis to discriminate between the effects of absolute concentrations of markers of the HPA axis and the RAAS (PC1: levels), and their relative concentrations (PC2: HPA axis dominance) on glucose metabolism. The results showed that HPA axis dominance i.e. cortisol/aldosterone ratio is significantly negatively correlated with insulin secretion (HOMA-β). Furthermore, the association between HPA axis dominance and insulin secretion remained significant after adjustment for multiple factors including reduced insulin sensitivity, which is known to induce compensatory insulin secretion. These results suggest that, although serum cortisol concentrations per se was also negatively associated with insulin secretion as we previously reported^27^, the cortisol/aldosterone ratio is a more fundamental and, thus, better indicator of risk for decreased insulin secretion in this general Japanese population.

These results may be of clinical importance to the development of diabetes therapies. For instance, inhibition of HSD1 can decrease the active steroid cortisol in target tissues (liver and adipose), suggesting it maybe an effective therapy for diabetes. However, although some studies have shown promising results, most programs developing HSD1 inhibitors have not produced sufficient good results and/or have been discontinued^28, 29^. Nevertheless, it is possible that the effects of these HSD1 inhibitors have not yet been suitable evaluated, as efficacy assessments can be difficult and have to be modified depending on class of drugs examined^30^. The results from the present study may be used to identify patients suitable for HSD1 inhibitor therapy i.e. those with higher cortisol/aldosterone ratio. Further, inhibition of GC action at the hypothalamus was shown to increase the anorexigenic properties of glucagon-like peptide-1 (GLP-1) treatment^15^. An anorexigenic or body weight loss effect of GLP-1 may thus be expected substantially in those with lower cortisol/aldosterone ratios, or may be effective if combined with an HSD1 inhibitor in those with higher cortisol/aldosterone ratios. Although these ideas are speculative, the issues appear to be clinically important and warrant further examinations in the future.

Since GCs and MCs are the effectors of the HPA axis and the RAAS, respectively, their concentrations represent not only their direct effect, but also the effects of the HPA axis and the RAAS. The HPA axis is a major stress response mediator that acts in conjunction with another major mediator, the autonomic nervous system (ANS). The HPA axis and the ANS are closely coupled and are often activated in parallel^31, 32^. Namely, higher cortisol/aldosterone ratios may also represent greater activation of the ANS. Accordingly, in conditions where the HPA axis activated, not only an increase in GC concentrations but also ANS activation may be involved in pathophysiology leading to diabetes^33^. Indeed, incubation of pancreatic islets with benextramine, a selective~ α2-adrenergic receptor agonist, has been shown to prevent the β-cell dysfunction of transgenic mice overexpressing the GC receptor specifically in β-cells^1^. Furthermore, β-cell dysfunction observed in mice treated with hydrocortisone was reported to be prevented by chlorisondamine (a ganglionic blocker) or phentolamine^34^. These findings indicate that association of higher serum cortisol concentrations with decreased β-cell dysfunction may be due, at least in part, to an activation of adrenergic signals. The RAAS is a hormonal cascade initiated by renin^35, 36^. Although aldosterone is the end molecule of the RAAS, aldosterone secretion is not only controlled by the RAAS but also by the HPA axis, as adrenocorticotropic hormone (ACTH) stimulates aldosterone secretion as well^37^. This means that plasma aldosterone concentration may represent both the RAAS and the HPA axis. We have previously examined the association between the HPA axis and the RAAS with regard to hypertension using PC analysis combining four hormones i.e. plasma renin activity (PRA), plasma aldosterone concentration, serum cortisol concentration and plasma ACTH concentration. The results of this study showed that serum cortisol concentrations and plasma aldosterone concentrations represent the HPA axis and the RAAS, respectively. On the basis of these findings, the results from the present study using PC analysis may not simply indicate association between these hormones and diabetes, but association between the HPA axis and The RAAS, and diabetes. In particular, our results show that HPA axis dominance over the RAAS is a significant marker for decreased insulin secretion, even in a general population. This finding suggests that those with HPA axis dominance over the RAAS are at risk of diabetes, even if individual hormone concentrations are within their physiological ranges.

A strength of the present study is what a relatively large population-based/general sample of individuals was used, and statistical adjustments were made for factors that could confound the results. In particular, the effects on insulin secretion were adjusted for reduced insulin sensitivity, which induces compensatory increases in insulin secretion. This is important because while previous studies on subjects with pathologically high serum cortisol concentrations have shown a positive correlation between serum cortisol concentrations and HOMA-β, their results may be confounded by the influence of compensatory increases in serum insulin with reduced insulin sensitivity^38–40^. Therefore, the results of these studies may not reflect the true effect of GC on insulin secretion. Namely, adjustments for reduced insulin sensitivity seem to be important to evaluate true association. When adjusting a statistical model for reduced insulin sensitivity, the reduced insulin sensitivity index, HOMA-R, may not be appropriate as a covariate for correlation analyses with the insulin secretion index, HOMA-β, since these indices are calculated using the same variables (FBG and IRI) and strongly correlated with each other. Namely, including HOMA-R and HOMA-β together in a multiple regression model may lead to misleading results becaouse of the fact, or multicollinearity. To avoid such multicollinearity, we measured CPR and used CRP-based reduced insulin sensitivity and secretion indices as covariates to ensure that statistical adjustments were appropriate for examining correlations with HOMA indices.

A limitation of the present study was that the subjects were participants in a health promotion study rather than an ordinary health check-up study, and may thus be more invested in keeping themselves healthy than the general population. Further, while we assumed that our subjects were relatively healthy, some of them may may have had modest pathological conditions such as subclinical Cushing syndrome, subclinical or partial adrenal insufficiency, or PA, as only basal serum cortisol concentrations and plasma aldosterone concentrations were analyzed in this study. Taken together, this means that the subjects may not accurately represent the general Japanese population. Further, as our study is cross-sectional and not a cohort study, we could not assess whether higher cortisol/aldosterone ratios are a risk for the future incidence of diabetes. Moreover, we did not exclude those taking drugs that can affect plasma aldosterone concentration (e.g. diuretics, angiotensin-converting enzyme inhibitors, angiotensin II type I receptor blockers, etc.). Most of subjects with hypertension in our study were on such drugs, and, thus, excluding subjects with hypertension from the study would have substantially reduced sample size. In addition, we believe that hypertensive subjects are at risk for abnormal glucose metabolism, and should be included in these kinds of studies. Finally, although indices (i.e. HOMA-β and CPI) we used are widely accepted as those representing insulin secretion, those do not appear to represent glucose-stimulated insulin secretion, since those are evaluated in fasting condition. Further, GCs administration has been shown to reduce insulin clearance via a reduction of hepatic activity of insulin-degrading enzyme, and, thus, increase serum insulin concentration.^41, 42^. Namely, GCs concentration may affect serum insulin concentration irrespectively of their effect on insulin secretion per se. Therefore, further studies with indices representing glucose-stimulated insulin secretion are awaited.

In conclusion, dominance of the HPA axis over the RAAS, as represented by the cortisol/aldosterone ratio, was significantly associated with decreased insulin secretion in a Japanese population. Although such association was observed with indices representing insulin secretion in a fasting condition, not with those representing glucose–stimulated insulin secretion, these results may inform further studies including those on how to best identify patients suitable for GC suppression therapies for metabolic disorders including diabetes. Further, our findings suggest that studies are also needed on whether a higher cortisol/aldosterone ratio is a risk factor for future incidence of diabetes.

## Methods

### Study population

Our subjects were recruited from the Iwaki study, a health promotion study of Japanese people over 20 years of age that aims to prevent lifestyle-related diseases and prolong lifespans. The study is conducted annually in the Iwaki area of the city of Hirosaki in Aomori Prefecture of Northern Japan^20, 43^. Of the 1,113 individuals enrolled in the Iwaki study in 2015, the following individuals were excluded from our study: 18 on drugs that appear to affect serum cortisol concentrations substantially (e.g. steroids and antidepressants), 4 with incomplete clinical data, 31 on medication for diabetes, and 44 with FBG levels below 63 mg/dl or over 140 mg/dl (to better evaluate HOMA indices). After these exclusions, 1,016 individuals (385 men, 631 women) aged 54.4 ± 15.1 years were included in our study.

This study was approved by the Ethics Committee of the Hirosaki University School of Medicine (No. 2014–014 and 2014–015), and was conducted according to the recommendations of the Declaration of Helsinki. Written informed consent was obtained from all participants.

### Characteristics measured

Blood samples were collected in the morning from peripheral veins of participants under fasting conditions in a supine position for 5 min after 10 min rest in a sitting position into 3 tubes (each one for measurements of whole blood, serum, and plasma, respectively), and values of characteristic were measured using an appropriate sample. For example, HbA1c levels were measred using a whole blood sample by enzymatic assay (CinQ HbA1c; Government Approval Number (GAN) 25A2 × 00001000008; Arkray, Inc., Kyoto, Japan), while blood glucose levels were measured using a serum sample with enzymatic/glucokinase-G6PDH assay (IatroLQ GLU; GAN 220AJAMX00004000; Unitika Ltd., Osaka, Japan). Serum levels of insulin (Architect insulin; GAN 12A2 × 00009000001; Abbott Japan Co., Ltd., Tokyo, Japan) and CPR (Chemilumi C-peptide; GAN 21300AMZ00465000; Siemens Healthcare Diagnostics K.K., Tokyo, Japan) were measure by chemiluminescent immunoassay. Accordingly, serum cortisol concentration was determined by chemiluminescent-enzyme-immunoassay (Access Cortisol Kit; GAN 20500AMY00118000; Beckman Coulter, Inc., Tokyo, Japan) and plasma aldosterone concentration by radioimmunoassay (SPAC-S Aldosterone Kit; GAN 16300AMZ00924000; Fujirebio Inc., Tokyo, Japan) according to the manufacturer’s instructions. Reduced insulin sensitivity and secretion were assessed by HOMA using FBG and insulin levels (HOMA-R and HOMA-β, respectively), which are defined by Matthews *et al*.^44^. The following clinical characteristics were also measured: height, body weight, BMI, HbA1c, systolic blood pressure, diastolic blood pressure, FBG, serum levels of insulin (IRI), total cholesterol, triglyceride (TG), HDL-cholesterol, uric acid (SUA), urea nitrogen, creatinine, and CPR. All laboratory testing were performed in a commercial laboratory (LSI Medience Co., Tokyo, Japan) according to the instructions of the venders. Reduced insulin sensitivity (IR(CPR)) and secretion (CPI) indices also assessed based on CPR concentrations, i.e. IR(CPR) = 20/(CPRxFBG)^45^ and CPI = 100xCPR/FBG, respectively, were used as covariates for adjustment. HbA_1c_ is expressed as the National Glycohemoglobin Standardization Program value. Diabetes was defined according to the 2010 Japan Diabetes Society criteria, i.e. FBG concentrations ≥ 126 mg/dl^21^. In subjects whose FBG levels were not measured, diabetes was defined as HbA1c levels ≥ 6.5%. None of the subjects in our study were known to have type 1 diabetes, and the number of subjects with diabetes was 40. Hypertension was defined as blood pressure ≥ 140/90 mmHg or taking treatment for hypertension (n = 385). Hyperlipidemia was defined as total cholesterol ≥ 220 mg/dl, TG ≥ 150 mg/dl or taking treatment for hyperlipidemia. (n = 475). Drinking alcohol (current or non-drinker) and smoking habits (never, past or current) were determined from questionnaires.

### Statistical analysis

Clinical characteristics are presented as means ± standard deviations (SD). The statistical significance of the difference in characteristics between two groups (parametric) and case-control associations between groups (nonparametric) was assessed by analysis of variance and χ^2^ tests, respectively. PC analysis was used to discriminate between the absolute concentrations of serum cortisol and plasma aldosterone as a whole (PC1: levels), and the ratio of serum cortisol concentrations to plasma aldosterone concentration (PC2: HPA axis dominance). Correlations between HOMA indices and clinical characteristics including PCs were assessed by linear regression analysis. Risk of higher PC2 score or cortisol/aldosterone ratio for decreased insulin secretion was evaluated by multiple logistic regression analysis with adjustment for factors found to be associated with insulin secretion by univariate regression analysis. Receiver operating characteristic (ROC) curve analysis was performed to determine cut-off values of the cortisol/aldosterone ratio to predict decreased insulin secretion. For statistical analyses, we log-transformed (log10) HOMA indices, serum cortisol concentration, plasma aldosterone concentration, cortisol/aldosterone ratio, CPI, and IR(CPR) to approximate a normal distribution. A value of p < 0.05 was accepted as statistically significant. PC and ROC curve analyses were performed using JMP software version 12.0.1(SAS Institute Inc., Cary, NC). All other analyses were performed using StatView software version 5.0 (SAS Institute Inc., Cary, NC).

## Electronic supplementary material

Supplementary Information
