# Supplementary material for: Dominance of the hypothalamus-pituitary-adrenal axis over the renin-angiotensin-aldosterone system is a risk factor for decreased insulin secretion
Source: Sci Rep. 2017 Sep 12;7:11360. doi: 10.1038/s41598-017-10815-y (PMC5596009; doi:10.1038/s41598-017-10815-y)
Supplement: Supplementary file 1 — Supplementary Information [file 41598_2017_10815_MOESM1_ESM.doc]

**Dominance of the hypothalamus-pituitary-adrenal axis over the renin-angiotensin-aldosterone system is a risk factor for decreased insulin secretion**

Makoto Daimon, Aya Kamba, Hiroshi Murakami, Satoru Mizushiri, Sho Osonoi, Kota Matsuki, Eri Sato, Jutaro Tanabe, Shinobu Takayasu, Yuki Matsuhashi, Miyuki Yanagimachi, Ken Terui, Kazunori Kageyama, Itoyo Tokuda, Shizuka Kurauchi, Shigeyuki Nakaji

Supplemenrary Table 1. Risk for decreased insulin secretion in those with normal FBG levels (<100mg/dl)

|  | | | |
| --- | --- | --- | --- |
|  | OR | 95％CI | p |
| per 1logF/PAC(ng) |  |  |  |
| Lower (ref) | 1 | - | - |
| Middle | 0.98 | 0.65-1.49 | 0.9379 |
| Higher | 1.82 | 1.20-2.76 | 0.0047** |
|  | | | |

#: Adjusted for multiple factors: age,gender, %fat, T-Cho, HDL, HbA1c, sBP, Alcohol, SUN, and IR(CPR).

Supplementary Table 2. Risk for decreased insulin secretion in each gender

|  | | | | | | | |
| --- | --- | --- | --- | --- | --- | --- | --- |
|  | Men | | |  | Women | | |
|  | OR | 95％CI | p |  | OR | 95％CI | p |
| per 1logF/PAC(ng) |  |  |  |  |  |  |  |
| Low er(ref) | 1 | - | - |  | 1 | - | - |
| Middle | 0.63 | 0.32-1.24 | 0.1787 |  | 1.42 | 0.87-2.33 | 0.1652 |
| Higher | 1.89 | 0.99-3.61 | 0.0536 |  | 2.06 | 1.23-3.45 | 0.0058** |
|  | | | | | | | |

#: Adjusted for multiple factors: age,gender, %fat, T-Cho, HDL, HbA1c, sBP, Alcohol, SUN, and IR(CPR).
